# Supplementary material for: UV222 disinfection of SARS-CoV-2 in solution
Source: Sci Rep. 2022 Aug 25;12:14545. doi: 10.1038/s41598-022-18385-4 (PMC9406255; doi:10.1038/s41598-022-18385-4)
Supplement: Supplementary file 1 — Supplementary Information. [file 41598_2022_18385_MOESM1_ESM.docx]

**UV_222_ disinfection of SARS-CoV-2 in solution**

# Authors

Richard T. Robinson^1,2^, Najmus Mahfooz^1^, Oscar Rosas-Mejia^1^, Yijing Liu^3^, Natalie M. Hull^3,4^*

# Affiliations

1. Department of Microbial Infection and Immunity, The Ohio State University, Columbus, OH, USA
2. Infectious Diseases Institute, The Ohio State University, Columbus, OH, USA
3. Department of Civil, Environmental, and Geodetic Engineering, The Ohio State University, Columbus, OH, USA
4. Sustainability Institute, The Ohio State University, Columbus, OH, USA

* Corresponding author: Natalie Hull, hull.305@osu.edu, 2070 Neil Ave, Hitchcock 417C, Columbus, OH,43210

# Contents

This supplementary information contains Figure S1 describing lamp emission and sample absorbance, Table S1 describing key parameters for UV exposures and dose calculations, Table S2 summarizing replication for the study, Table S3 summarizing amplification in negative controls, Figure S2 containing representative plaque assay results, Figure S3 showing standard curves for qPCR and ELISA assays, Figure S4 showing N gene damage immediately after UV treatment (Day 0) at dose 0 -10 mJ/cm^2^, and Figure S5 showing molecular assay concentration data for N gene or N protein at each UV dose.

**Figure S1:** (A) The raw spectral emission from 200 - 300 nm of the filtered excilamp (USHIO Care222^®^) was interpolated and relativized to the peak emission at 222 nm for use in UV dose calculations and plotted on log scale to show orders of magnitude less but non-zero emission at filtered wavelengths > 240 nm. (B) The absorbance spectrum from 200 - 300 nm of SARS-CoV-2 at ~10^5^ PFU/mL in cDMEM was measured for each of three Tests for use in UV dose calculations.

**Table S1:** Summary of key UV dose calculation parameters for each independent Test.

|  | Test 1 | Test 2 | Test 3 |
| --- | --- | --- | --- |
| Date | 1-Sep-20 | 16-Sep-20 | 4-Nov-20 |
| UV doses (mJ/cm^2^) | 0, 0.7, 1.1, 1.4, 1.7, 2.0, 2.7 | 0, 10, 16, 20, 25, 30, 40 | 0, 2, 4, 8, 12, 16, 20, 30 |
| Sample exposure times (sec) | 0, 29, 45, 57, 72, 87, 115 | 0, 214, 343, 429, 536, 643, 856 | 0, 84, 168, 336, 504, 672, 840, 1260 |
| Incident irradiance at center of petri dish (mW/cm^2^) | 1.100 | 2.490 | 1.200 |
| Divergence factor | 0.9444 | 0.9091 | 0.9091 |
| Petri factor | 0.9459 | 0.9147 | 0.9791 |
| Water factor | 0.0247 | 0.0232 | 0.0230 |
| Average irradiance through sample depth (mW/cm^2^) | 0.0236 | 0.0466 | 0.0238 |

**Table S2:** Summary of biological and technical replicates for all the culture and molecular assays.

| Assay | Biological Replicates | Technical Replicate (Day 0 Samples) | Technical Replicate (Day 3 Samples) |
| --- | --- | --- | --- |
| SARS-CoV-2 plaque assay | 3 Independent UV disinfection tests (test 1, test 2 and test 3) | Test 1:2 | Test 1:2 |
|  |  | Test 2:2 | Test 2:2 |
|  |  | Test 3:2 | Test 3:2 |
| SARS-CoV-2 N gene quantitation N1 primer set | 3 Independent UV disinfection tests (test 1, test 2 and test 3) | Test 1:1 | Test 1:2 |
|  |  | Test 2:2 | Test 2:2 |
|  |  | Test 3:2 | Test 3:2 |
| SARS-CoV-2 N gene quantitation N1-2 primer set | 3 Independent UV disinfection tests (test 1, test 2 and test 3) | Test 1:2 | Test 1: N/A |
|  |  | Test 2:2 | Test 2: N/A |
|  |  | Test 3:2 | Test 3: N/A |
| SARS-CoV-2 N protein ELISA | 3 Independent UV disinfection tests (test 1, test 2 and test 3) | Test 1:2 | Test 1:2 |
|  |  | Test 2:2 | Test 2:4 |
|  |  | Test 3:2 | Test 3:4 |

Note: For the technical replicate Day 3 samples with ELISA assay, the original sample value that outside the standard curve were diluted further and rerun as a replicate. We had 3 dilution series.

| **Virus titer (PFU/mL)** | **UV Dose (mJ/cm^2^)** | **Plaque assay** |
| --- | --- | --- |
| 0 | 0 | 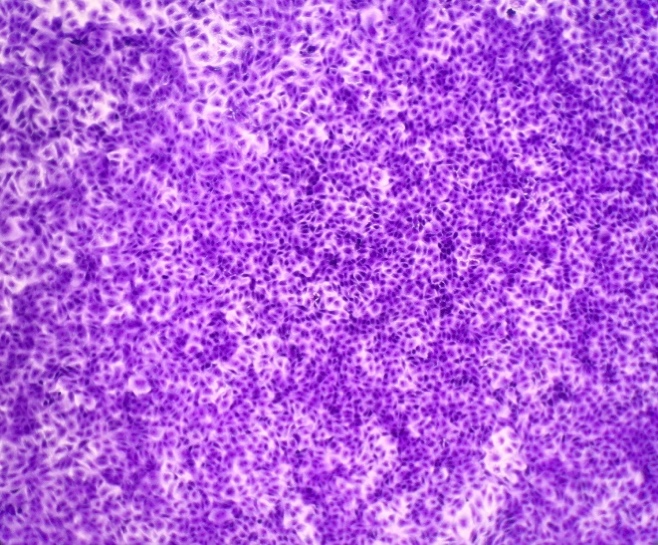 |
| 10^6^ | 0 | 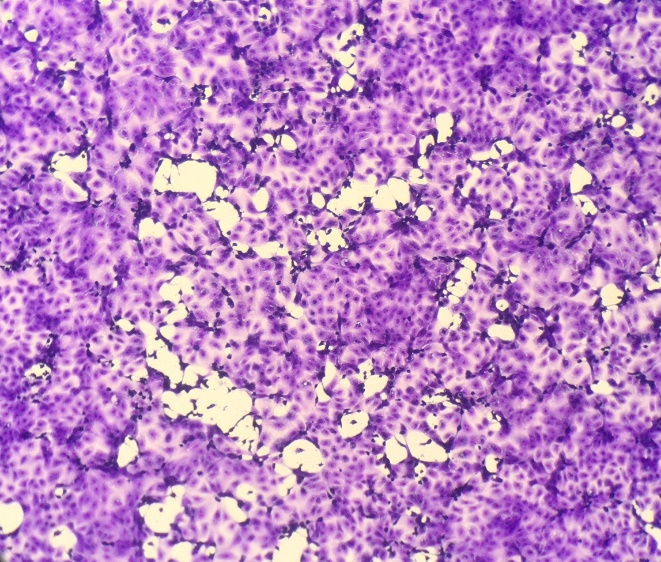 |
| 10^6^ | 40 | 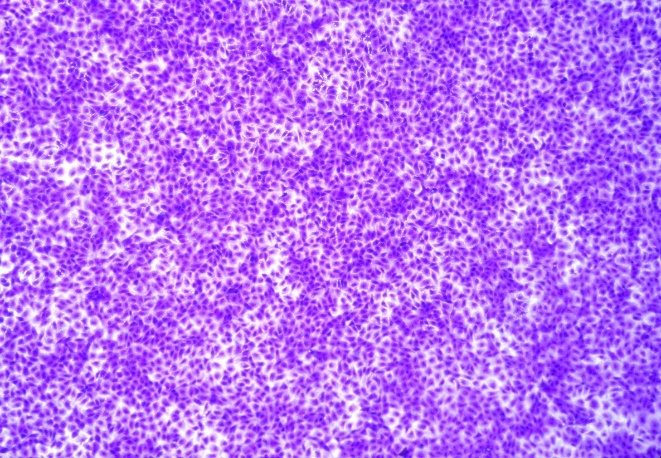 |

**Figure S2:** Representative plaque assay results from Experiment 2.

**Table S3**: Summary of CT values for RNA extraction negative controls in three independent UV disinfection tests

| N1 Assay | RNA extraction negative controls CT Values |
| --- | --- |
| Test 1 | 30.87 |
| Test 2 | 32.8 |
| Test 3 | 34.21 |


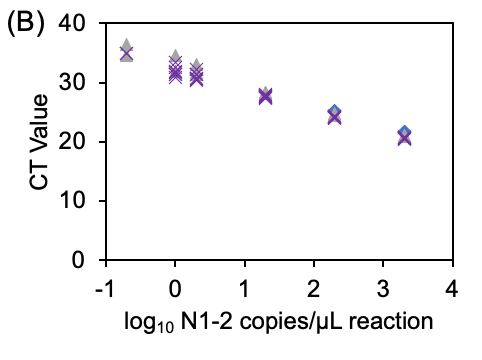


**Figure S3:** N gene RT-qPCR standard curves gene copies/µL reaction for the (A) short N1 amplicon and (B) long N1-2 amplicon and (C) ELISA N protein standard curve. Colors differentiate individual assay runs.

**Figure S4:** SARS-CoV-2 N gene damage immediately after UV treatment (Day 0) expressed as log_10_ reduction of N1 (short amplicon) copies/µL in qPCR reactions.

(A)

**Figure S5:** N gene RT-qPCR copies/µL reaction for the (A) short N1 amplicon and (B) long N1-2 amplicon, where error bars represent standard deviation of at least two technical replicates and could include technical replicates averaged across dilutions. (C) N protein ELISA pg/mL, where error bars represent standard deviation of at least two technical replicates and could include technical replicates averaged across dilutions. Day 0 samples were analyzed immediately after UV irradiation, where Day 3 samples were analyzed in culture supernatants after incubation of samples with host cells.
